# P060: Surveillance and control of community-associated methicillin-resistant Staphylococcus aureus in Geneva, Switzerland, 2002 – 2012

**DOI:** 10.1186/2047-2994-2-S1-P60

**Published:** 2013-06-20

**Authors:** A Huttner, E Von Dach, N Liassine, M Descombes, R Auckenthaler, G Renzi, P François, S Harbarth, P Sudre

**Affiliations:** 1 HUG, Geneva, Switzerland; 2 Dianalabs, Geneva, Switzerland; 3 Unilabs, Geneva, Switzerland; 4 Synlab, Geneva, Switzerland; 5 DGS, Geneva, Switzerland

## Introduction

Community-associated methicillin-resistant *Staphylococcus aureus* (CMRSA) was first reported in Geneva in 2002; a prospective surveillance system based on voluntary reporting was then established.

## Objectives

We report trends in CMRSA infections in Geneva Canton from 2002 through 2012.

## Methods

The Cantonal Antibiotic Resistance Group, representing Geneva’s Department of Health, University Hospitals and public and private laboratories, defines a CMRSA case as (1) a clinical infection from which MRSA is isolated in any adult or child with (2) no hospitalizations in the previous 12 months (excluding hospital stays for infants at birth), and (3) residence in Geneva Canton. Four laboratories as well as physicians from public hospitals and private practice participate. For each laboratory-reported case, a questionnaire is sent to the clinician for demographic and clinical information. Contact tracing is performed to detect clustering. Descriptive analyses were performed using Stata 12.

## Results

There were 245 CMRSA cases in the 11-year surveillance period; the mean attack rate was 4.9 cases/100K inhabitants. Median age at diagnosis was 31 years (interquartile range, 13–48); 20% were under ten. Skin infections dominated (82%). There were no cases of necrotizing pneumonia, fasciitis or infection-related death. Incident cases predominated (75%); recurrences did not increase over time. Most infections (75%) occurred in persons without comorbidity; “chronic dermatologic condition” was the most common comorbidity (15/48, 31%) followed by diabetes mellitus (8/48, 17%). Fifty clusters were identified, most being family-related. After peaking at 8.4 cases/100K inhabitants in 2005, CMRSA incidence has since plateaued at a mean of 5.5 cases/100K inhabitants.

## Conclusion

After peaking in 2005, CMRSA infections appear to have stabilized despite continuous introduction of new strains. The surveillance system and related measures appear to be successful in containing transmission in Geneva Canton.

## Disclosure of interest

None declared

